# A matrix-based method of moments for fitting the multivariate random effects model for meta-analysis and meta-regression

**DOI:** 10.1002/bimj.201200152

**Published:** 2013-02-08

**Authors:** Dan Jackson, Ian R White, Richard D Riley

**Affiliations:** 1 MRC Biostatistics UnitCambridge, CB2 0SR, UK; 2 School of Health and Population Sciences, University of BirminghamUK

**Keywords:** Kronecker product, Meta-regression, Method of moments, Multivariate meta-analysis

## Abstract

Multivariate meta-analysis is becoming more commonly used. Methods for fitting the multivariate random effects model include maximum likelihood, restricted maximum likelihood, Bayesian estimation and multivariate generalisations of the standard univariate method of moments. Here, we provide a new multivariate method of moments for estimating the between-study covariance matrix with the properties that (1) it allows for either complete or incomplete outcomes and (2) it allows for covariates through meta-regression. Further, for complete data, it is invariant to linear transformations. Our method reduces to the usual univariate method of moments, proposed by DerSimonian and Laird, in a single dimension. We illustrate our method and compare it with some of the alternatives using a simulation study and a real example.

## 1 Introduction

Multivariate meta-analysis is a fairly recent methodological development (e.g. van Houwelingen et al., 1993, 2002; Berkey et al., 1998), which is becoming more commonly applied in medical statistics (Jackson et al., 2011). Multivariate meta-analysis is used to synthesise multiple outcome effects from separate studies (e.g. overall and disease free survival), whilst allowing for their correlation. Two types of correlations may exist: *within-study correlations*, which indicate the association between outcome effect estimates in each study, and *between-study correlations*, which indicate how the true outcome effects are associated across studies. The within-study correlations arise when the same patients contribute data to both outcomes in a study. The between-study correlation arises when (unknown) factors causing between-study heterogeneity induce a correlation in the true outcome effects across studies; for example studies with a larger than average treatment effect on overall survival will typically have a larger than average treatment effect on disease free survival.

Multivariate meta-analysis possesses many advantages over its more established univariate counterpart, including the potential for inferences for different outcomes to ‘borrow strength’ (Riley et al., 2007) from each other. Jackson et al. (2011) discuss the advantages, and limitations, of multivariate compared to univariate meta-analysis. Software has been produced in Stata to fit the random effects meta-analysis model (White, 2009), and has recently been extended to multivariate meta-regression models (White, 2011), and the R package mvmeta (Gasparrini, 2011) is now available.

Here, we take the multivariate random effects model as the standard model. The fixed effect model assumes that common underlying effects apply to all studies. We find this generally implausible: it is a very strong assumption to assume that there is no between-study heterogeneity in any of the outcomes included in the analysis. When fitting the multivariate random effects meta-analysis model, however, we must estimate the between-study covariance matrix, which increases the computational demands. We assume that within-study covariance matrices are available for all studies but recognise that obtaining the within-study correlations is often a practical difficulty and that these values are important (Riley, 2009). See Jackson et al. (2011) for a variety of methods for handling unknown within-study correlations and Riley et al. (2008) for an alternative random effects model that does not require them.

Several fully parametric approaches to estimation have been developed. These include maximum likelihood, restricted maximum likelihood (REML; e.g. van Houwelingen et al., 2002; Jackson et al., 2011) and Bayesian estimation (Nam et al., 2003). Maximum likelihood methods are invariant to linear transformations but, especially in high dimensions, are much more computationally intensive.

Semi-parametric alternatives therefore have their advantages, such as the method based on *U* statistics (Ma and Mazumdar, 2011). The method proposed by DerSimonian and Laird 1986 has also been extended to the multivariate setting (Jackson et al., 2010; Chen et al., 2012). By estimating the between-study covariance matrix by matching moments a valid, but not optimal, analysis may be performed without requiring the assumption of between-study normality. The more general validity of the non-likelihood-based methods may be considered advantageous because we can only invoke the Central Limit Theorem to justify this assumption by the notion that the unobserved random effects are the sum of several different factors. Despite this lack of optimality, the simulation studies performed by Ma and Mazumdar 2011, Jackson et al. 2010 and Chen et al. (2012) suggest that the semi-parametric methods perform well compared with likelihood-based methods when making inferences about the treatment effect. However, the method proposed by Jackson et al. 2010 is not invariant to linear transformations and the procedure described by Chen et al. (2012) cannot handle covariates or missing outcome data. Since missing outcome data are a very common occurrence, it is vitally important that estimation procedures handle them in an appropriate way. The aim of this paper is to provide a new estimation method that overcomes the problems associated with the existing methodologies.

This paper presents a multivariate generalisation of DerSimonian and Laird’s extremely popular univariate method. The new method can handle missing data and can adjust for covariates in a meta-regression, and reduces to the method of Chen et al. (2012) with complete data and no covariates. Like the method by Chen et al. (2012), the new method is based on matrix operations and is invariant to linear transformations. The rest of the paper is set out as follows. In Section 2, we present our new method and derive its properties. In Section 3, we present some results from a simulation study and in Section 4, we apply our methods to an example. We conclude with a discussion in Section 5.

## 2 A new method of moments for multivariate meta-analysis and meta-regression

We present the general case for random effects multivariate meta-regression, and so include meta-analysis as a special case where there are no study level covariates and intercepts alone are included in the model. We let *n* and *d* denote the number of studies and the dimension (the number of study outcomes under consideration) of the meta-analysis or meta-regression, respectively.

The multivariate random effects meta-regression model (Jackson et al. 2011; White 2011) is
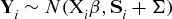
(1)for all studies 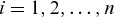, where 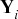 is the 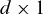 column vector of outcomes (or summary effect measures) associated with study *i*, S_*i*_ is the 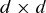 corresponding within-study covariance matrix, Σ is the 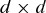 between-study covariance matrix, X_*i*_ is the 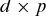 design matrix for study *i* and 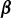 is the 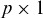 column vector containing the true effects. For a multivariate meta-analysis (no covariates), X_*i*_ is the 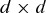 identity matrix and 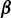 is the 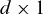 column vector of average outcome effects. If instead, covariate effects are included then the design matrix X_*i*_ contains further columns of covariates in order to describe the multivariate meta-regression. We adopt the convention of treating the entries of S_*i*_ as fixed constants but these quantities are estimated in practice. If a study does not provide all outcomes then, assuming these are Missing at Random (MAR), the model for the outcomes for study *i* is taken as the marginal model from (1).

The estimate of Σ is of direct interest because this describes the correlations between the outcomes and quantifies the between-study heterogeneity. Once Σ has been estimated, however, the standard procedure for making inferences about 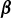, which contains the parameters of primary interest, assumes 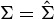 (Jackson et al., 2011). This approximation is justified provided that there is a sufficiently large number of studies. This eases the computation because, once both the within and between-study covariance matrices are regarded as known, all the vectors of outcomes 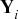 are treated as normally distributed with fixed and known covariance matrices. Inference then proceeds as a weighted linear regression were all weights are known. We adopt this standard procedure when implementing our methodology below so that the only computational difficulty to overcome is the estimation of Σ.

### 2.1 Two Q matrices for multivariate meta-analysis and meta-regression

We begin by fitting the fixed effect model, that is (1) with 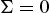, so that the residuals from this model can be used to estimate the between-study covariance matrix. The fixed effects model assumes that there is no between study heterogeneity and is computationally straightforward to fit using generalised least squares because all within-study covariance matrices are regarded as known. We then obtain the fitted 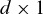 outcome vectors from this model, which we denote by 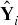; this includes the fitted values for any missing components of 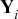. If there are no covariates then the fitted outcome vectors for all studies are given by the fixed effect pooled estimates, for example.

Having obtained these fitted outcome vectors, we define our first 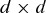 Q matrix as
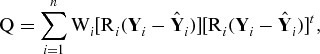
(2)where *t* denotes matrix transpose, R_*i*_ is a 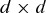 diagonal matrix containing the missing data indicator of 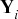; the *j*th entry of the leading diagonal of R_*i*_ is equal to one if 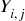 is observed and is zero if 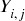 is missing. W_*i*_ is the 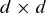 within-study precision matrix associated with study *i*. If all outcomes are provided by study *i* then 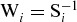 but if some outcomes are missing then we compute the rows and columns of S_*i*_ corresponding to the outcomes that are available and obtain the inverse of the resulting matrix of reduced dimension. Then we obtain W_*i*_ by including columns and rows of zero that correspond to the unobserved outcomes whilst the other rows and columns of W_*i*_ are given by the corresponding entries of 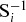.

The pre-multiplication of the residuals by R_*i*_ in (2) ensures that those corresponding to missing outcomes do not contribute to Q; the entries of 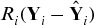 corresponding to missing outcomes are zero. Hence missing entries of the 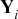 vectors may be replaced by zero, or any other arbitrary value, when computing Q. 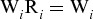 and 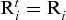 so that Q can be more conveniently evaluated as
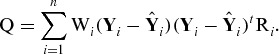
(3)

Our second Q matrix is Q^*t*^, so that
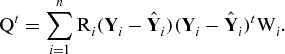


Both Q and Q^*t*^ simplify to Cochran’s *Q* statistic in the context of a univariate meta-analysis and to its established analogue in the context of a univariate meta-regression. That is, in the more usual univariate notation (DerSimonian and Laird, 1986), for a univariate meta-analysis
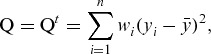
where the 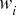 are the reciprocals of the within-study variances and 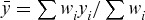. Since the fixed effect fitted outcome vectors are obtained without iteration, computing Q and Q^*t*^ also does not require any iteration.

An alternative and also natural Q matrix, of the form suggested by Jackson et al. 2010 is given by
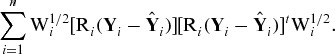
Another possibility is to use
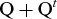
as the Q matrix. These matrices give rise to estimating equations that are similar to the ones that follow in both form and derivation. However, the invariance property derived below in Section 2.5 also does not apply when using these alternatives. Hence we prefer to use the proposed Q in Equation (3), its transpose Q^*t*^ and the procedure that follows, to these possibilities.

### 2.2 The expectation of Q and Q^*t*^

Following Jackson et al. 2010, we will use the method of moments to estimate Σ. In order to evaluate the expectation of Q, and hence Q^*t*^ and ultimately estimate Σ, we vertically stack the 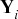 into a single 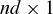 column vector **Y**, where any missing entries are replaced by zero or any other arbitrary value, and define a corresponding block diagonal 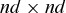 precision matrix W. Here, the *i*-th sub-matrix along the block diagonal of W is W_*i*_. We define the 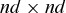 matrix R = diag(R_*i*_), which we take as a fixed constant, and we show in Appendix that
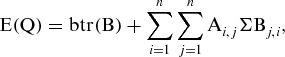
(4)where
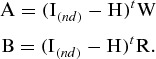



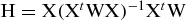
, X denotes the matrix produced by vertically stacking the X_*i*_, and 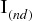 denotes the 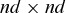 identity matrix. Here, we partition the 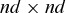 matrices A and B into *n*^2^ blocks of dimension 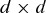 and in (4) and (5) we denote the *i*-th by *j*-th sub-matrix of A and B by 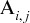 and 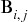, respectively. We use the notation btr(B) to denote the ‘block-trace operator’ of the 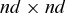 matrix B, defined as the sum of the *n* sub-matrices of dimension 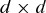 along the main diagonal of B. The dimension of btr(B) is therefore 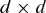. Because Σ is symmetric, it immediately follows that
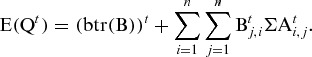
(5)

### 2.3 Obtaining estimates of Σ by matching moments

Equations (4) and (5) can be used to provide two alternative estimates of Σ but we will see in the next section that these are very closely related. For estimation purposes we replace E(Q) and E(Q^*t*^) with their observed values, and Σ with its estimate, so that, for example (4) becomes
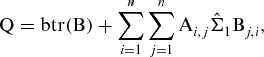
(6)and we solve for 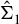, which is sandwiched between the 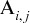 and 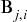 terms. In order to make progress, we use the vec matrix operator, where vec(M) denotes the column vector created by stacking the columns of M, and the identity vec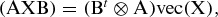 where ⊗ denotes the Kronecker product (Henderson and Searle, 1981. Applying the vec operator and this identity to (6) gives
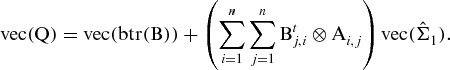
(7)Equation (7) can then be solved for 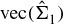 and hence 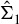.

The estimating Equation (7) makes it clear that the estimation procedure results in a system of *d*^2^ simultaneous equations for the *d*^2^ entries of Σ. However, Σ is a symmetric matrix which means that (7) provides a single estimate for the diagonal entries (the between-study variances) but two estimates of each of the off-diagonal entries (the between-study variances). A natural solution to resolving the difficulty of having pairs of estimates of the between-study covariances is to average them. This is exactly what we ultimately do, but we justify this by using Q^*t*^ to provide another system of simultaneous estimating equations as explained below. For now, however, we have an interim estimate 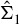 from (7), which is asymmetrical.

If 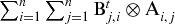 is singular, then estimation using (7) fails, which indicates that the comparison of the magnitude of Q to its expected value is insufficient to result in *d*^2^ linearly independent equations. This is appropriate in extreme cases where there are insufficient data to fit the model in this way. For example, in the case of a multivariate meta-analysis (no covariates), where all studies provide all outcomes, and where all studies’ within-study covariance matrices are identity matrices, 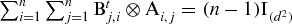. Hence, the estimation fails when 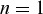, but otherwise estimates are obtained. Since there is no information about the between-study variation when we have just a single study, it is appropriate that the estimation should fail in such instances.

Similarly (5) results in
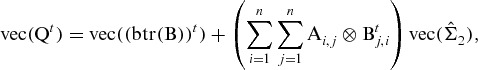
(8)which, assuming that the estimation does not fail because of insufficient data, can be solved for 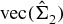 and hence 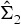 can be obtained. 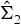 is a second interim estimate that is not symmetrical.

### 2.4 The relationship between the two estimates of Σ and a final estimate of Σ

Equations (7) and (8) give rise to estimates 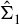 and 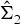, respectively, but it is easily shown that these estimates are very closely related. Let P_*d*_ denote the particular permutation matrix for 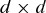 matrices with the following two properties (Henderson and Searle 1981, their Equations (5) and (25), respectively):
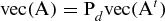
and
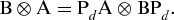


Then by pre-multiplying both sides of (8) by P_*d*_, replacing vec(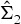) with 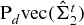 and making use of the above two properties immediately yields (7) where 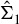 has been replaced by 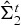. We therefore deduce that 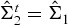. A simple way to obtain a symmetric matrix from a non-symmetric matrix A is to calculate the sum 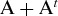. Hence, by taking the average of the two estimates
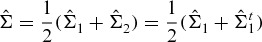
(9)

we arrive at a symmetrical, but not necessarily positive semi-definite, 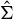. This is equivalent to averaging the pairs of estimates of the between-study covariances that result from (7), or equivalently these pairs from (8). Both the estimates in these pairs estimate the same between-study covariances, so in large samples the estimate from (9) will approximately solve both (7) and (8).

To address the fact that 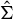 is not necessarily positive semi-definite, we write 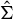 in terms of its spectral decomposition
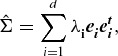
where 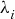 is the *i*-th eigenvalue of 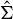 and 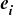 is the corresponding normalised eigenvector. We suggest using
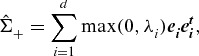
to produce a ‘truncated’ symmetric and positive semi-definite estimate of Σ. This procedure reduces to the univariate method of DerSimonian and Laird, and the corresponding method of moments for meta-regression, in a single dimension.

### 2.5 Invariance properties of the estimator for complete data

If the data are complete, so that all components of 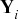 are observed, (3) becomes
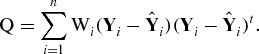
(10)

Suppose we apply a non-singular linear transformation C to our data prior to analysis, so that the transformed data are 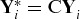, 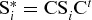 and 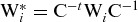, where 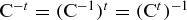. Then calculating Q* using the transformed data, and comparing with (10), we see that 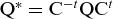, so that 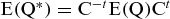. Hence, when we equate 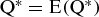, when producing the estimate 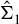, this is equivalent to solving 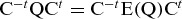, which can be expressed as
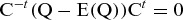
so that the solution of the estimating equation also satisfies Q = E(Q), the estimating equation prior to transforming the data. More directly, if there are complete data then 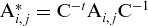 and 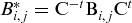, so that 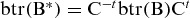. Writing (6) in terms of the transformed quantities, and using these identities with 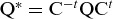, almost immediately yields 
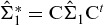
. Similar observations apply to 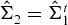.

Therefore, the ‘untruncated’ 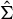 possesses a highly desirable invariance property if the data are complete: we obtain the same result if we analyse the data and then transform the estimate, or transform the data and then perform the estimation. Since 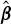 depends only on the estimated variance structure, this estimate also possesses this invariance property if no truncation of 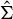 is required. The previously proposed method of moments by Jackson et al. 2010 does *not* possess this property, however, a point we illustrate numerically using our example in Section 4.

Finally, *if there are no covariates so that we have complete data in the context of a multivariate meta-analysis*, then the formulae for E(Q) and E(Q^*t*^) simplify. Defining 
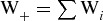
, for example (4) becomes
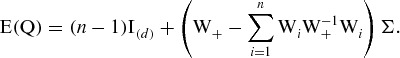
(11)

This is a more obvious generalisation of the usual univariate result (DerSimonian and Laird, 1986 and can be equated to the observed Q and solved without using the vec operator. Solving (11) to obtain 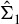, and using our proposed estimate (9), immediately yields the estimator suggested by Chen et al. (2012). Hence, our methodology is a more general version of theirs, where our proposal can also handle missing outcome data and covariates.

### 2.6 Making inferences about the average outcome effects vector β

Having estimated the between-study variance matrix, inference for 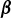 proceeds by taking 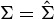 and therefore weighted linear regression where all weights are known (Jackson et al., 2010; White, 2011). Let 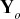 denote the stacked vector of the observed entries of 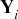, let X_*o*_ denote its design matrix and let 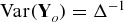, where Δ is treated as fixed and known. then
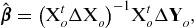
which is approximately normally distributed with covariance matrix
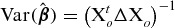
so that standard errors of the estimates can be obtained as the square root of the diagonal entries of 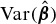. Ninety-five per cent confidence intervals can be obtained as the estimates plus and minus 1.96 standard errors. This procedure was used to calculate confidence intervals in the simulation study in Section 3, but quantiles from the *t*-distribution are sometimes used for this purpose (Jackson et al., 2010.

## 3 Simulation study

In order to compare the proposed method to some of the alternatives, the simulation study by Jackson et al. 2010 was extended using R (R Development Core Team, 2012). Initially 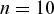 and 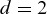 was used, without including any covariates, which provide a moderate number of studies and a two-dimensional multivariate meta-analysis.

For each simulation, two sets of 10 within-study variances were simulated from 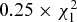, but values outside the range (0.009, 0.6) were discarded. These two sets of within-study variances were then ranked, and the first study was taken to have the largest pair of simulated variances, and so on, until the last study had the smallest pair of simulated within-study variances. New within-study variances were simulated for every meta-analysis in the simulation study. Study outcomes were simulated from model (1) using means of zero, although this choice is immaterial. Between-study variances of 0, 0.024 and 0.168 were used because these values correspond to *I*^2^ statistics (the proportion of total variation in the outcomes that is due to between-study heterogeneity) of 0, 0.3 and 0.75, respectively (Jackson et al., 2010). Within-and between-study correlations of 0, 0.7 and 0.95 were used. The between-study variance matrix for each simulated dataset was then estimated using the proposed method, the previously proposed multivariate DerSimonian and Laird procedure (Jackson et al., 2010) and REML. Inferences for β were also made using the three methods, in particular the proportion of nominal 95% confidence intervals for the first entry of 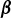, that contain the true value of zero were compared where these intervals were computed as described in Section 2.6. A total of 1000 simulations were used for each simulation run.

Some results from the simulation study are shown in Table 1, where we show the results that we consider to be of primary importance. We show the estimates of the between-study variance and the coverage probability of confidence intervals for the first outcome only, but these results for the second outcome can be ascertained from other simulation runs and symmetry. Table 1 shows that the proposed method performs very similarly to the previously proposed methods on average.

**Table 1:** Some results from the simulation study with 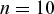 and complete data, where 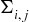 denotes the *i*-th row and *j*-th column of Σ and ρ denotes the within-study correlation (assumed constant across studies). In each case ‘Proposed’, ‘Previous’ and ‘REML’ denote values using the proposed method, the previous multivariate DerSimonian and Laird method (Jackson et al., 2010) and the REML procedure, respectively. E(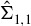) denotes the average estimated between-study variance for the first outcome and E(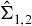) denotes the average estimate of the between-study covariance. Coverage is the proportion of nominal 95% confidence intervals for the first entry of 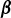 that contain the true value zero

					E( 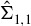 )			E( 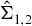 )			Coverage		
Run	Σ_1, 1_	Σ_2, 2_	Σ_1, 2_	ρ	Proposed	Previous	REML	Proposed	Previous	REML	Proposed	Previous	REML
1.	0	0	0	0	0.018	0.018	0.015	0.000	0.000	0.003	0.961	0.961	0.960
2.	0	0.024	0	0	0.020	0.020	0.016	0.000	0.000	0.004	0.960	0.960	0.952
3.	0	0.168	0	0	0.018	0.018	0.017	0.002	0.002	0.005	0.962	0.962	0.965
4.	0.024	0	0	0	0.038	0.038	0.037	0.000	0.000	0.004	0.936	0.936	0.928
5.	0.024	0.024	0	0	0.035	0.035	0.033	0.000	0.000	0.003	0.941	0.941	0.925
6.	0.024	0.168	0	0	0.037	0.037	0.037	0.002	0.002	0.005	0.927	0.929	0.919
7.	0.168	0	0	0	0.166	0.166	0.167	0.000	0.000	0.005	0.912	0.912	0.913
8.	0.168	0.024	0	0	0.167	0.167	0.167	0.000	0.000	0.001	0.895	0.895	0.892
9.	0.168	0.168	0	0	0.168	0.168	0.167	−0.001	−0.001	0.000	0.915	0.916	0.915
10.	0.024	0.024	0.017	0.7	0.035	0.035	0.037	0.021	0.021	0.025	0.930	0.927	0.925
11.	0.024	0.168	0.045	0.7	0.035	0.035	0.036	0.045	0.044	0.052	0.919	0.919	0.925
12.	0.168	0.024	0.045	0.7	0.177	0.176	0.179	0.049	0.048	0.054	0.904	0.908	0.914
13.	0.168	0.168	0.118	0.7	0.169	0.170	0.172	0.116	0.117	0.119	0.891	0.892	0.885
14.	0.024	0.024	0.023	0.95	0.033	0.035	0.035	0.029	0.030	0.032	0.910	0.909	0.911
15.	0.024	0.168	0.060	0.95	0.039	0.035	0.037	0.060	0.061	0.070	0.934	0.938	0.949
16.	0.168	0.024	0.060	0.95	0.175	0.171	0.183	0.067	0.062	0.075	0.889	0.889	0.905
17.	0.168	0.168	0.160	0.95	0.170	0.171	0.173	0.160	0.160	0.164	0.898	0.890	0.893

### 3.1 Further results and simulation studies

A very thorough simulation study, examining six different scenarios, was performed: (1) the situation considered above with 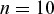 and complete data; (2) 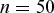 and complete data; (3) 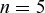 and complete data; (4) a *t*-distribution for the random effect and complete data; (5) missing data where half of the first outcomes are missing completely at random; (6) meta-regression. In addition to the results shown in Table 1, for all scenarios we calculated the number of times the two methods of moments required truncating, the Monte Carlo error of the estimated effects and the empirical standard error of the estimated variance components. We also extended the simulation study to include further runs using the same parameter values as runs 10–17, but instead using within-study correlations of zero, to mimic meta-analyses of diagnostic test accuracy. All these additional results, and the results in Table 1, are available in the Supporting Information that accompanies this paper.

The results in the Supporting Information show that all three methods generally perform very similarly on average. However, a few interesting conclusions can be drawn from these results, for example the asymptotic efficiency of the REML estimates of the variance components can be seen in the results for 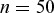, but this more precise estimation does not appear to provide better inference for the pooled estimates. The necessity to truncate moments based estimators was usually a very rare event when 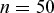 and between-study heterogeneity was considerable (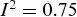) for both outcomes. The only exception to this was in the final run where, to mimic diagnostic test accuracy studies, the within-study correlation was zero but the between-study correlation was 0.95. This is perhaps something of an extreme case, where the two outcomes of interest are quite highly correlated but there is no within-study correlation. Evidently, without any within-study correlation to explain the often highly correlated simulated outcomes, the two methods of moments required truncating much more often than might be anticipated on the basis of the large sample size and the considerable marginal between-study variances.

The results for 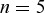 suggest that this sample size is too small to accurately apply all three methods because coverage probabilities of nominal 95% confidence intervals in the range 0.85–0.90 were quite common. However there is no evidence of bias in the pooled estimates, even when data are missing. REML performed well when the random effects model is misspecified using a *t*-distribution; Ma and Mazumdar 2011 found that this was also the case for other random effects distributions. Finally, the two methods of moments generally provided very similar rates of requiring truncation to ensure a positive semi-definite estimated between-study covariance matrix, but the proposed method required truncating more often when covariate effects were included in the final simulation study where a multivariate meta-regression model was used.

To summarise, the results from the simulation studies reassure us that the proposed method generally performs very similarly to the established methods on average and so is a viable alternative. This is also the conclusion of Chen et al. (2012), whose method is equivalent to ours when there are no covariates and no missing data, who consider alternative parameter values in their simulation study. However, differences can occur for particular datasets as our example in the next section shows.

## 4 Example: Treatment for hypertension

We illustrate our method using a real example. The method has been implemented in the Stata software mvmeta (White 2009; White 2011) which is available by typing net from http://www.mrc-bsu.cam.ac.uk/IW_Stata/ within Stata. This example involves 10 studies that assess the effectiveness of hypertension treatment for lowering blood pressure. Each study provides complete data on two treatment effects, the difference in systolic blood pressure (SBP) and diastolic blood pressure (DBP) between the treatment and the control groups, where these differences are adjusted for the participants’ baseline blood pressures. A bigger reduction in blood pressure is a desirable outcome, so negative estimates indicate that the treatment is beneficial. The within-study correlations are known, so that the within-study covariance matrices are also known (Riley et al., 2008a), and the data are shown in Table 2.

**Table 2:** Data from 10 studies that assess the effectiveness of hypertension treatment for lowering blood pressure. SBP and DBP are the treatment effects on the systolic and diastolic blood pressures, respectively. The within-study standard error corresponding to each estimate is given in parentheses and the within-study correlations are denoted by ρ. Negative estimates indicate that the treatment is beneficial. Isolated systolic hypertension (ISH) is an indicator for the inclusion of ISH patients only

Study	SBP	DBP	ρ	ISH
1.	−6.66 (0.72)	−2.99 (0.27)	0.78	0
2.	−14.17 (4.73)	−7.87 (1.44)	0.45	0
3.	−12.88 (10.31)	−6.01 (1.77)	0.59	0
4.	−8.71 (0.30)	−5.11 (0.10)	0.77	0
5.	−8.70 (0.14)	−4.64 (0.05)	0.66	0
6.	−10.60 (0.58)	−5.56 (0.18)	0.49	0
7.	−11.36 (0.30)	−3.98 (0.27)	0.50	0
8.	−17.93 (5.82)	−6.54 (1.31)	0.61	1
9.	−6.55 (0.41)	−2.08 (0.11)	0.45	1
10.	−10.26 (0.20)	−3.49 (0.04)	0.51	1

The results using the proposed method, and the previously proposed method of moments and REML, are shown in Table 3. REML provides larger estimates of the between-study variances and so results in larger standard errors for the outcome vector parameters, but we have strong evidence that the treatment is beneficial for both outcomes.

**Table 3:** Results from the multivariate meta-analysis. The estimates are shown using the proposed method, the previously proposed method of moments (Previous method of moments (MM); Jackson et al., 2010) and REML. Standard errors for the parameters included in 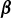 are shown in parentheses

Parameter	Proposed method	Previous MM	REML
β_1_ (SBP)	−9.17 (0.55)	−9.13 (0.54)	−9.50 (0.77)
β_2_ (DBP)	−4.31 (0.36)	−4.30 (0.36)	−4.43 (0.48)
Σ_1, 1_	2.03	1.95	3.92
Σ_1, 2_	0.20	0.06	1.81
Σ_2, 2_	1.05	1.03	1.83

In order to illustrate the invariance property possessed by our proposed method, we also performed the analysis in terms of the two outcomes SBP-DBP (pulse pressure) and DBP. In the notation used in Section 2.5, this corresponds to the transformation
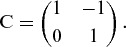


REML and (because the data are complete and no ‘truncation’ was required to provide a positive semi-definite between-study covariance matrix) the proposed method provides results that are invariant to this transformation, that is 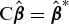 and 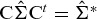. However, as expected, the previously proposed method of moments by Jackson et al. 2010 does not provide invariant results, despite also not requiring truncation. For example, this method gives
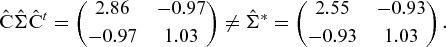


Fortunately, for this example, this lack of invariance does not have much impact on inferences for the treatment effect parameters.

### 4.1 A multivariate meta-regression to investigate the implications of isolated systolic hypertension

Three studies (studies 8–10, see Table 2) involve only subjects with isolated systolic hypertension (subjects with high SBP, but normal DBP). We might therefore anticipate that the treatment effect will be different in these trials. In particular we might expect the treatment, which appears to be generally effective, to be less effective for DBP in these three trials. This is because there is less scope for the treatment to be effective for this outcome and type of subject, because their DBP is less extreme to begin with. In order to test the hypothesis that the treatment effects are different in these trials, the indicator that the trial includes only ISH patients was included as a covariate for both outcomes in a bivariate meta-regression.

The estimated regression coefficients associated with ISH are shown in Table 4. REML provides larger estimates of between-study variance (results not shown) and so provides larger standard errors than the moments based methods. The overall picture from Table 4 is that, because of the large and positive estimated ISH coefficients associated with DBP, trials that include only ISH patients provide smaller DBP treatment effects, as anticipated. However, the statistical significance of this conclusion is sensitive to the estimation method used.

**Table 4:** Results from the multivariate meta-regression. The estimated regression coefficients associated with ISH are shown using the proposed method, the previously proposed method of moments (Previous MM; Jackson et al., 2010) and REML. Standard errors are shown in parentheses

ISH regression coefficient	Proposed method	Previous MM	REML
SBP	0.46 (1.56)	0.48 (1.41)	0.23 (1.87)
DBP	1.52 (0.57)	1.49 (0.61)	1.36 (0.95)

## 5 Discussion

We have developed a matrix-based multivariate extension of DerSimonian and Laird’s univariate method. By handling both missing data and covariates, our method also extends the method proposed by Chen et al. (2012). The moments-based estimator of the between-study covariance matrix that we have developed possesses a desirable invariance property with complete data. The proposed method of truncation does not preserve this property when it is used to ensure that the estimated between-study covariance matrix is positive semi-definite, however. Likelihood-based methods, including REML, possess good invariance properties, but these come at the price of being fully parametric and computationally intensive. If a method for truncation could be developed, which preserves the invariance property of the ‘untruncated’ estimate, then this might be considered preferable and this is currently being investigated. Despite this, our proposed method of moments retains most of the advantages of the other semi-parametric procedures: it is non-iterative, fast and, because the between-study covariance matrix is estimated by matching moments, does not require the assumption of between-study normality. However, it is not quite as transparent as its predecessors and it requires more sophisticated matrix operations. Like its predecessors, since it does not take into account the uncertainty in the estimated between-study covariance matrix, the proposed method requires a reasonable number of studies in order to provide accurate inferences; for example, our simulation study suggests that 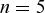 is too small even if there are no missing outcome data. The method can be used for any dimension of multivariate meta-analysis, but the available data may place constraints on what is appropriate. If binary data are modelled using normal approximations in model (1) and the outcome is rare, then inferential procedures that use the binomial distribution directly are more appropriate. The proposed method does not currently incorporate methods based on generalised linear mixed models, but this provides a possible avenue for further work. Furthermore, the proposed method has not been shown to possess any optimality properties, rather it has been derived as a natural and easily implemented multivariate extension of one of the most popular univariate methods used in meta-analysis.

Although an advantage of the semi-parametric methods is that they require weaker assumptions than those based on likelihood based methods, they also have their limitations. For example, reduced models for the random effect, where perhaps all between-study correlations or variances are assumed to be the same across outcomes, may be fitted using likelihood-based methods by adding these constraints when performing the numerical maximisation. It is much less obvious how to impose these constraints when using the method of moments. Reduced models for the random effect may be needed to identify models with limited amounts of data and this is an important issue for further research. Quantifying the uncertainty in the estimated between-study covariance matrix may also be of interest and this may require some form of bootstrapping when the method of moments has been used. This too requires further investigation.

We have applied our method to a variety of real examples. A large sample empirical investigation examining its use compared to the various alternatives is of interest and may form the subject of future work. In our experience, alternative estimation methods provide similar results across meta-analyses as a whole, but can provide markedly different results for particular meta-analytic datasets. Examples where the inferences resulting from alternative estimation methods differ are of interest and may help us to better understand the features of data that result in this. A variety of multivariate estimation methods are now available to the meta-analyst, so an assessment of the sensitivity of the model fit to the procedure used may easily be performed. If very marked differences are obtained using different estimation methods, then the reasons for this should be investigated, and these are most likely to occur when there are insufficient data available to adequately identify the random effects model.

In conclusion, we feel that we have produced a useful and computationally straightforward method for multivariate meta-analysis and meta-regression. We propose that our method is, at the very least, a useful addition to the existing methodologies.

## Appendix

In this Appendix, we derive the expectation of Q by forming a single model that describes the data from all studies. We then evaluate the expectation of a larger matrix from which we can conveniently evaluate E(Q) as given in Equation (4).

We vertically stack the , with any missing values of the  replaced by zero (or any other arbitrary value) into a single vector **Y** and define a corresponding block diagonal precision matrix W_*p*_; W_*p*_ is matrix W where any zero main diagonal entries are replaced by *p*;  denotes the variance attributed to the imputed zeros. The random effects model (1) for the imputed data is

(A.1)

where X denotes the matrix produced by vertically stacking the X_*i*_, I_(*n*)_ denotes the *n* by *n* identity matrix;  is just a block diagonal matrix with *n* blocks of Σ. We recover model (1) for the original data as  but do not set  in model (A.1) because we make use of . It is a standard result that

(A.2)

where  is the fitted outcome vector obtained when fitting the fixed-effect model, that is model (A.1) with . By direct evaluation and observation, we have that

(A.3)

and

(A.4)

that is  is idempotent. We define the matrix

(A.5)

where R is a diagonal matrix containing the missing data indicator of **Y**. We have  and , so that  and . From (A.1) and (A.2) we have that

so that, treating R as a constant and taking the expectation of (A.5), and upon making further use of (A.2), gives

(A.6)

Combining (A.6) with the variance of **Y** in (A.1) gives

so that, using (A.3) and (A.4), and then taking the limit ,

where

and

Noting that W and R are block diagonal, we have that

so that

Recalling that  is a block diagonal matrix of *n* blocks of Σ, we have that

so that

as given in Equation (4). An alternative Q matrix, of the form suggested by Jackson et al. 2010, is given by

If we define

then the expectation of this alternative Q matrix can be shown to be

in a very similar way.

## Notes

As a service to our authors and readers, this journal provides supporting information supplied by the authors. Such materials are peer reviewed and may be re-organized for online delivery, but are not copy-edited or typeset. Technical support issues arising from supporting information (other than missing files) should be addressed to the authors.
