# Supplementary material for: A matrix-based method of moments for fitting the multivariate random effects model for meta-analysis and meta-regression
Source: Biom J. 2013 Feb 8;55(2):231–45. doi: 10.1002/bimj.201200152 (PMC3806037; doi:10.1002/bimj.201200152)
Supplement: Supplementary file 1 [file bimj0055-0231-sd1.pdf]

Simulation Study

For each of six scenarios, up to 25 simulation runs were performed, where the Run number refers to the parameter values shown in the table below. The first 17 runs correspond to those shown in table 1 of the paper and 18-25 repeat runs 10-17 but the within-study correlations are set to 0, in order to mimic studies of diagnostic test accuracy. 1000 bivariate datasets were simulated in each scenario for all runs and three methods were applied to the resulting datasets: the proposed method, REML and the previously proposed method of moments. The simulation study was performed using R software. The R spaces created to perform the simulation study are available from the first author on request.

Run	$\Sigma_{1,1}$	$\Sigma_{2,2}$	$\Sigma_{1,2}$	ρ
1	0	0.004	0	0
2	0	0.024	0	0
3	0	0.168	0	0
4	0.024	0	0	0
5	0.024	0.024	0	0
6	0.024	0.168	0	0
7	0.168	0	0	0
8	0.168	0.024	0	0
9	0.168	0.168	0	0
10	0.024	0.024	0.017	0.7
11	0.024	0.168	0.045	0.7
12	0.168	0.024	0.045	0.7
13	0.168	0.168	0.118	0.7
14	0.024	0.024	0.023	0.95
15	0.024	0.168	0.06	0.95
16	0.168	0.024	0.06	0.95
17	0.168	0.168	0.16	0.95
18	0.024	0.024	0.017	0
19	0.024	0.168	0.045	0
20	0.168	0.024	0.045	0
21	0.168	0.168	0.118	0
22	0.024	0.024	0.023	0
23	0.024	0.168	0.06	0
24	0.168	0.024	0.06	0
25	0.168	0.168	0.16	0

Scenario 1: Complete data with n=10 studies

This is the scenario explored in table 1 of the paper. Here we provide more results in the spreadsheet “1. 10 STUDIES WITH COMPLETE DATA”.

In this spreadsheet we show, for all three estimation methods:

Column B: The mean estimate of the first treatment effect (or outcome).
Column C: The MC error of the mean estimate of the first treatment effect.
Column D: The mean estimate of the between-study variance of the first treatment effect.

Column E: The empirical standard deviation of the estimate of the between-study variance of the first treatment effect.
Column F: The mean estimate of the between-study covariance.
Column G: The empirical standard deviation of the estimate of the between-study covariance.
Column H: The estimated coverage probability of the confidence interval for the first treatment effect.
Column I: The number of times (out of 1000) that the estimated between-study covariance matrix was truncated when using the two methods of moments.

Results for the parameters relating to the second treatment effect may be obtained by symmetry.

Scenario 2: Complete data with n=50 studies

As scenario 1 but now n=50.

Scenario 3: Complete data with n=5 studies

As scenarios 1 and 2, but now n=5.

Scenario 4: Complete data with n=10 studies using a bivariate t-distribution to simulate the between-study heterogeneity

This is as scenario 1 but now we use the “mnormt” R package to simulate the random effects distribution for the between-study heterogeneity using a multivariate t distribution. This means that model (1) is now incorrectly specified and the assumptions made by the REML analysis are violated.

We simulate the studies’ within-study variability using a bivariate normal distribution with covariance matrix equal to the studies’ within-study covariance matrices, but now simulate the between-study variability using a bivariate t distribution with 4 degrees of freedom. Here the “Scale matrix”, **S**, for the bivariate t distribution is half the between-study covariance matrix (see the mnormt documentation, in general **S** * dof/(dof-2) equals the covariance matrix). We centre both the within and between-study variance structures at zero but this choice is immaterial.

Four degrees of freedom was chosen to provide heavy tails but finite 1st, 2nd and 3rd order central moments, so that the random effects distribution for the heterogeneity is not very severe. When using the bivariate t distribution we only simulated datasets where there is some heterogeneity in both outcomes. This is because a heavy tailed random effects distribution for the heterogeneity for one outcome, and no heterogeneity at all for the other, seems implausible. Hence we omitted runs 1-3 and 7 when using the bivariate t distribution in this way.

Scenario 5: Incomplete data with n=10 studies

As scenario 1 but now half of the studies (chosen randomly) do not provide estimates of the first treatment effect, where these data are missing completely at random (MCAR).

In the spreadsheet "5. 10 STUDIES WITH INCOMPLETE DATA" we show, for all three estimation methods:

Columns B-E: The mean estimates of the treatment effects and the MC errors.
Columns F-K: The estimated between-study variance matrix parameters and the empirical standard deviations of these estimates.
Columns L-M: The estimated coverage probabilities of the confidence intervals for the treatment effects.
Column N: The number of times (out of 1000) that the estimated between-study covariance matrix was truncated when using the two methods of moments.

Scenario 6: Meta-regression with n=10 studies

As scenario 1 but now we introduce a covariate where the 1st, 3rd, 5th, 7th and 9th studies provide a covariate of 0 and the other studies provide a covariate of 1. This covariate structure was chosen in order to mimic a covariate describing the type of study, where there are two equally likely study types.

In the spreadsheet "6. 10 STUDIES & META-REGRESSION" we show the results in a similar way as in the previous scenario but instead of the treatment effect parameters we show the corresponding results for the two covariate effect parameters, ***whose true values are one and two for the first and second treatment effects respectively***.
